# Genome-Wide Gene Expression Analysis Suggests an Important Role of Suppressed Immunity in Pathogenesis of Kashin-Beck Disease

**DOI:** 10.1371/journal.pone.0028439

**Published:** 2012-01-03

**Authors:** Shuang Wang, Xiong Guo, Xiao-ming Wu, Mikko J. Lammi

**Affiliations:** 1 Department of Orthodontics, Stomatological Hospital, Key Laboratory of Environment and Genes Related to Diseases, Department of Public Health, College of Medicine, Xi'an Jiaotong University, Ministry of Education, Xi'an, Shaanxi, P.R. China; 2 The Key Laboratory of Biomedical Information Engineering of Ministry of Education, School of Life Science and Technology, Xi'an Jiaotong University, Xi'an, P.R.China; 3 Department of Biosciences, University of Eastern Finland, Kuopio, Finland; University of Georgia, United States of America

## Abstract

**Objective:**

To investigate the differences between the gene expression profiles in peripheral blood mononuclear cells (PBMC) from normal controls and patients with Kashin-Beck disease (KBD).

**Methods:**

Twenty KBD patients and 12 normal subjects were selected from a KBD-endemic area and divided into four pairs of KBD vs. control (KBD, n = 5 per pair; control, n = 3 per pair). RNAs were respectively isolated from KBD PBMCs and normal PBMCs. Gene expression profiles were analyzed by oligonucleotide microarray. The gene expression profiles in PBMCs from KBD patients and normal controls were compared and the differentially expressed genes were identified. The obtained microarray data was further confirmed by using quantitative real-time reverse transcription polymerase chain reaction (qRT-PCR).

**Results:**

Approximately 501 genes, corresponding to 2.4% of the total probe transcripts, showed a 2-fold change in differential expression. 19.4% (97 out of 501)of the differentially expressed genes were commonly detected in all the four pairs. Among the 97 differentially expressed genes, 83 genes were up-regulated and 14 genes were down-regulated, compared with those in the normal controls. Some differentially expressed genes were found to be related to functions such as immunity, metabolism, apoptosis, cystoskeleton and cell movement, and extracellular matrix. The validity of our microarray data were supported by the results of qRT-PCR assay.

**Conclusion:**

Differences in the PBMC gene expression profile between the KBD patients and the normal controls exhibited a similar pattern among all the four pairs of microarrays examined, indicating that the suppressed immunity may play an important role in the pathogenesis of KBD.

## Introduction

Kashin-Beck Disease (KBD) is a chronic, endemic osteochondropathy with unknown etiology. The disease is mainly distributed in a diagonal belt ranging from the northeast to the southwest of China, where the selenium content is low in the soil. Over 2.5 million patients are affected by the disease, and approximately 30 million people are at risk [1]. Recent investigations have shown a high prevalence of the disease in Western China. The KBD prevalence rates in children aged 7 to 13 years old are as high as 50.43%, 27.36%∼32.93% and 11.34%∼23.08% in the monitoring sites in Tibet, Qinghai and Shaanxi provinces, respectively [2]. Two major environmental hypotheses have been proposed for the pathogenesis of KBD: 1) endemic selenium deficiency, and 2) serious cereal contamination by mycotoxin-producing fungi [3], [4]. Clinically, the disease is manifested by arthritic pain, morning stiffness, enlarged and shortened fingers, deformed and enlarged joints, and limited motion of the joints in the extremities [2]. The seriously affected children may suffer from shortened stature or dwarfism, and disability in their daily life.

The basic pathological feature of KBD is focal chondronecrosis in the deep zones of the growth plate cartilage and articular cartilage, which can result in impaired endochondral development, secondary osteoarthritis and disability in the advanced stages. Besides chondrocyte necrosis, apoptosis and dedifferentiation of chondrocytes and abnormal expressions of collagen types I, II, III, VI and X in the articular cartilage of KBD patients have been reported [5], [6], [7]. Cellular factors, such as Bcl-2, TGF-beta, bFGF and PTHrP, are also abnormally expressed in the KBD articular cartilage [8]. In both KBD and non-KBD areas, KBD patients have higher serum nitric oxide synthase (NOS) and inducible NOS levels than normal controls [9], [10]. This indicates that the nitric oxide (NO) pathway may play a role in the regulation of chondrocyte necrosis. Significant differences in D12S367 and D12S1638 loci on chromosome 12 have been observed between KBD patients and normal controls by gene scan technique [11].Those studies indicate that expressions of some genes may be changed in KBD patients; however, no systemic studies have been done on the differences between the gene expression profiles of KBD patients and normal controls.

In this study, Agilent Human 1A Oligo microarray (V2) analysis was used to compare the PBMC gene expression profiles of the KBD patients and the normal controls. The validity of the obtained oligonucleotide array data was evaluated by the parallel analyses of selected transcripts using quantitative real-time reverse transcription polymerase chain reaction (qRT-PCR).

## Materials and Methods

### Group division and disease diagnosis

This study was approved by the Human Ethics Committee of Xi'an Jiaotong University.Adult subjects were randomly chosen from Nanshao village of Yongshou county, Shaanxi province, China. Nanshao village is a KBD-endemic area with an adult prevalence rate of 38.24%. KBD patients were diagnosed based on National diagnostic criteria of Kashin-Beck disease in China [GB16395-1996]. Controls and KBD patients with a history of any other bone and joint diseases were excluded from the study. According to the exclusion and enrolment criteria for KBD, 20 KBD patients and 12 normal subjects were selected for the study, and divided into four pairs of KBD vs. control (KBD, n = 5 per pair; control, n = 3 per pair), with their ages, genders and areas matched (Table 1). Both KBD and control subjects were of Chinese Han lineage. All subjects provided a written informed consent.

**Table 1:** KBD/control sample pairs used for microarray analysis.

	KBD				Control			
Sample pair	n	Age, years (range)	Male	Female	n	Age, years (range)	Male	Female
1	5	47.20(41–55)	2	3	3	45.00(35–63)	1	2
2	5	50.40(43–58)	2	3	3	48.67(39–68)	1	2
3	5	50.20(40–67)	2	3	3	44.67(37–59)	1	2
4	5	46.60(38–58)	3	2	3	41.00(27–54)	0	3
total	20	48.60(38–67)	9	11	12	44.84(27–68)	3	9

### Blood collection and peripheral blood mononuclear cell isolation

Three milliliters of peripheral blood was collected from each subject into heparinized vacutainer tubes (Becton Dickenson, San Jose, California, USA) for gene expression analysis. Leukocyte cell numbers were determined using a Hemovet 950 (Drew Scientific, Oxford, Connecticut, USA). PBMCs were isolate from the plasma by centrifuging blood at 1500×g for 20 minutes. The cell pellet was resuspended in Hanks' balanced salt solution (Gibco BRL/Invitrogen, Carlsbad, California, USA) and the cell suspension was carefully layered over 5 ml of Lympholyte-H (Cedarlane Labs, Hornby, Ontario, Canada) in a 15 ml Falcon tube and centrifuged for 40 minutes at 1500×g. Then the leukocyte layer was collected using a Pasteur pipette. The cells were rinsed twice with cold Hanks' balanced salt solution and stored in RNAlater (Ambion Inc., Austin, Texas, USA) until RNA isolation.

### cRNA preparation and microarray hybridization

The total RNA was isolated from PBMCs using Trizol reagent (Life Technologies Inc., Carlsbad, California, USA) and was used to generate cRNA probes for microarray hybridization. The concentrations and qualities of all RNA samples were determined using a high resolution electrophoresis system (Agilent 2100 bioanalyzer, Agilent Technologies, Palo Alto, California, USA). Degraded and/or contaminated RNA samples were identified by visual inspection of the electropherograms produced by the Aglient 2100 bioanalyzer. The presence of distinct 18S and 28S ribosomal peaks and the absence of multiple peaks corresponding to smaller RNA fragments were indicative of high-quality RNA samples. To avoid individual differences among subjects, 30 µl of the total RNA was extracted from each individual subject and then pooled to form 4 pairs of microarrays, as shown Table 1.

cRNA preparation and microarray hybridization and scanning were performed according to the manufacturer's protocol (Agilent Technologies). Briefly, 2 µg of total KBD or control RNA in each pair was reverse transcribed into cDNA, which was further transcribed into cRNA and labeled with CyDye, using Amino Allyl MessageAmp aRNA Kit (Ambion) according to the manufacturer's protocol. Before reverse transcription, the RNA was treated with RNase-free DNase I (Ambion) to remove residual genomic DNA. After that, 0.5 µg of the labeled control and KBD cRNA in each pair was purified, and mixed with hybridization buffer before being applied to the microarrays.

The PBMC gene expression profiles of the KBD patients and the normal controls were compared by Agilent Human 1A Oligo microarray (V2) analysis. Agilent's Human 1A Oligo microarray (V2) consists of 21073 (60-mer) oligonucleotide probes, which span conserved exons across the transcripts of the targeted full-length genes. In this study, a two-channel labeling system was used to hybridize two differently labeled RNA samples to the same microarray. The control cRNA in each pair were labeled with Cy5 while the KBD cRNA were labeled with Cy3. The cRNA was hybridized to microarrays for 17 hr at 65°C in the presence of hybridization buffer according to the Agilent protocol. The microarray slides were then washed. Immediately after the wash buffer was removed by centrifugation, the microarray slides were scanned using Gene-Pix 4000B (Axon Instruments Inc., Foster City, California, USA), which used a 532nm laser for Cy3 measurement and a 635nm laser for Cy5 measurement.

### Normalization and analysis of microarray data

Sixteen-bit TIFF images produced by the Axon scanner were analyzed using the GenePixPro 3.0 software package (Axon Instruments Inc.). After the Cy3 and Cy5 grayscale images were obtained, each pseudo-color image was overlaid, and all spots in the ratio image were defined by accessing the gene list file that described the location of each gene on the microarray. The average signal intensity was subtracted from the median background intensity and was output with the UniGene and GenBank descriptors to a Microsoft Excel data spreadsheet. Relative expression levels were calculated by global normalization between two samples using all detected genes. A more detailed description of this nomalization process can be accessed from www.aglient.com. Individual genes were classified as “up-regulated” or “down-regulated” when the fold change of spot labeling intensity for a pair of transcripts was more than twofold or less than 0.5-fold, respectively. The “fold change” value represents the ratio of the signal intensity of the KBD sample to the signal intensity of the control sample in each pair. Significant differences in gene expressions between the KDB and control samples were determined by the *t*-test. *p* values were calculated using the standard combinatorial approach and then adjusted for multiple testing using the Bonferroni method. The Bonferroni corrected *p*-value of 0.01 was considered statistically significant.We have deposited the raw data at GEO under accession number GSE32127, we can confirm all details are MIAME compliant.

### Quantitative real-time reverse transcription PCR

Five of the genes found to be up-regulated in the microarray analysis were chosen to be validated by qRT-PCR using total RNA samples from additional 20 subjects (n = 10 for KBD; n = 10 for control). The 2^−ΔΔCt^ relative quantification method was applied for the relative quantification in gene expression and β-actin was used as an internal control. (Table 2). β-actin was chosen as the normalization reference for gene expressions across samples.

**Table 2:** KBD/control sample pairs used for qRT-PCR.

	KBD		Control	
Sample pair	Age, years	Sex	Age, years	Sex
1	42.3	Female	46.4	Female
2	49.2	Female	47.6	Female
3	55.6	Female	54.3	Female
4	46.8	Male	46.5	Male
5	52.1	Male	53.3	Male

Total RNA was prepared for qRT-PCR assay in the same way as for the oligonucleotide array analysis. Purified RNA was reversely transcribed into cDNA using Superscript II RT (Invitrogen, Karlsruhe, Germany). An equivalent amount of RNA was added to the reaction mix in addition to 12.5 ml SYBR Green (Applied Biosystems, Foster City, CA, USA) and 0.5ml forward and reverse primers (10 pmol/ml) (MWG-Biotech, Ebersberg, Germany). Nuclease-free water was also added so that the final volume per well was 25 ml. Primer sequences are listed in Table 3. qRT-PCR reactions were performed using the ABI7500 Real-Time PCR system (Applied Biosystems) according to the manufacturer's instructions. The reactions were incubated at 94°C for 2 min for 1 cycle, and then at 94°C (30 seconds), 53°C (30 seconds) and 72°C (30 seconds) for 45 cycles. All PCR reactions were confirmed to be valid by the presence of a single peak in the melt curve analysis and the presence of a single specific product in electrophoresis on a 2.5% weight/vol agarose gel. Relative fold change of each individual gene was calculated using the comparative Ct equation (User Bulletin #2, 2001, Applied Biosystems) as follows: 2^−ΔΔCt^ where ΔΔCt = ΔCt (KBD sample) − mean ΔCt (control sample); ΔCt = Ct (target gene) − Ct (housekeeping gene), where Ct values of target genes were normalized to Ct values of housekeeping gene (β-actin). Reactions were performed in triplicate.

**Table 3:** Primers used in qRT-PCR validation of microarray data.

Symbol	5′ Primer sequence	3′ Primer sequence	Amplicon size (bp)
BMPR1A	CGA AGA TAT GCG TGA GGT TGT G	GTC TGG AGG CTG GAT TGT GG	135
ADAM28	GGA ATT GGG AGA GGA CTG TGA TTG	TCA GGC AGG TCG CAC TCA TC	126
CCR4	GCC AGT GTC AGG AGG AAG	GGT GTG AGG AAG GAT GCC	144
IGLL1	CCT CGG TCA CTC TGT TCC	CTT GTT GTT GCT CTG TTT GG	125
DUOX1	TGA CAG ATG TGC CAG ATA CC	TGA CGG ATG ACT TGA GAG CC	131
β-ACTIN	TGC GTG ACA TTA AGG GAG AG	AGG AAG GAA GGC TGG AAG	120

## Results

### Findings of the genome-wide screening

The PBMC gene expression profiles of the KBD patients and the normal controls were compared by using the Agilent Human 1A high density oligonucleotide array system, which covers 21073 human genes. The qualities of all total RNA samples were determined by electrophoresis. The presence of distinct 18S and 28S ribosomal peaks and the absence of multiple peaks corresponding to smaller RNA fragments were detected, which indicated that the total RNA samples were of high quality. Thus, the microarray results from these RNA sample pools are considered to be reliable.

**Figure 1:**
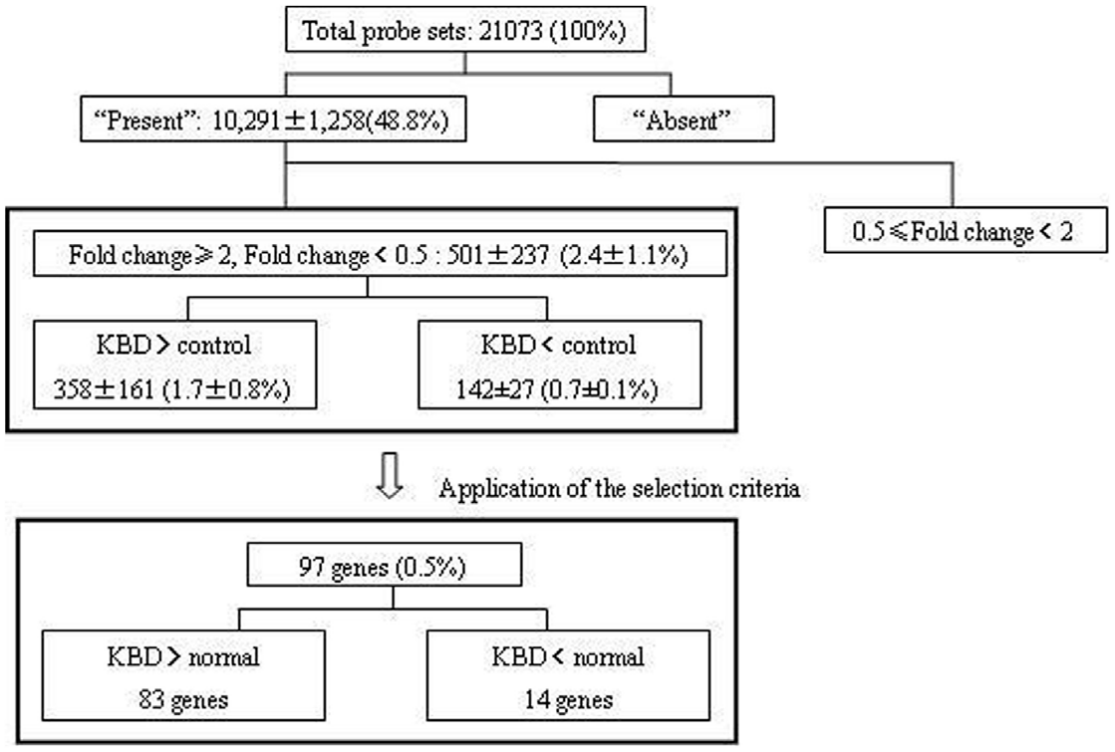
Flow chart for the identification of the differentially expressed genes in PBMCs of KBD patients and healthy controls. The terms “present” and “absent” represent the expression levels of the transcripts described in the Materials and Methods section. Values are the mean±SD number and percentage of transcripts. See Materials and Methods for details of the selection criteria.

Genome-wide expression analysis detected ≈49% (mean±SD 10,291±1,258 transcripts) of all probe sets corresponding to the transcripts present in each PBMC sample (Figure 1). However, the KBD patients and normal controls showed slight difference in the percentages of these transcripts expressed in PBMCs:52.2% for KBD patients vs 46.8% for normal controls.

A more than 2-fold difference in the transcript gene expression between the KBD patients and healthy controls in each pair was noticed in 2.4±1.1% (501±237 genes) of all probe sets. 1.7±0.8% of the transcripts (358±161 genes) showed high expression levels while 0.7±0.1% of the transcripts (142±27 genes) showed low expression levels in the KBD samples, compared with the transcripts in the control samples. Furthermore, 19.4% (97 out of 501)of the differentially expressed genes were commonly detected in all four pairs.

Thus, a total of 97 genes were identified to be differentially expressed. Eighty-three of them were up-regulated and 14 of them down-regulated in KBD PBMCs, compared with those in the control PBMCs. These genes were then subdivided into functional categories. The up-regulated genes were subdivided into 17 categories, including immunity related, receptor, metabolism, apoptosis, cystoskeleton and cell movement, DNA modification, oncogene related, protein synthesis and modification, development, ion channel transport protein, extracellular matrix related, cytokine factor, cell cycle, Zinc finger protein, signal transduction, and transcription related. The down-regulated genes were subdivided into 11 categories, including receptor, cystoskeleton and cell movement, extracellular matrix, metabolism, cytokine factor, protein synthesis and modification, ion channel transport protein, signal transduction, growth factor and haematogenesis. All the down-regulated genes and part of the up-regulated genes are presented in Table S1 and Table 4, respectively.

**Table 4:** List of down-regulated genes in KBD PBMC.

Category	Gene name	Public ID	Gene symbol	Fold change Mean±SEM
Receptor	Protein with high similarity to an odorant receptor	AB065913.1	OR4D1	0.42±0.02
Cystoskeleton and cell movement	Protein with high similarity to actin-binding LIM protein 1	NM_014945.1	ABLIM3	0.43±0.06
Extracellular matrix related	Retired, was Member of the keratin B2 high sulfur family	NM_033059.2	KRTAP4-14	0.34±0.03
Metabolism	Homo sapiens phospholipase A2 delta	NM_178034.1	PLA2G4D	0.38±0.02
Cytokine factor	Protein containing five fibronectin type III domains	NM_020962.1	NOPE	0.37±0.01
	Precursor of peptide YY	D13899.1	PYY	0.32±0.02
Protein synthesis and modification	Vaccinia related kinase 2	BC027854.1	VRK2	0.36±0.05
Ion channel transport protein	Protein containing a pleckstrin homology (PH) domain	NM_018071.1	FLJ10357	0.41±0.02
Signal transduction	Phosphatidylinositol 3-kinase class 3	Z46973.1	PIK3C3	0.34±0.08
Growth factor	Neurotrophin 3	M37763.1	NTF3	0.26±0.01
Ionophorous transport protein transmembrane protein related	Chondrolectin	AF257472.1	CHODL	0.25±0.02
Haematogenesis	Hemoglobin beta subunit	BC007075.1	HBB	0.46±0.02
Miscellaneous	Protein of unknown function	NM_014129.1	FLJ90396	0.43±0.05
	Retired, was Protein of unknown function	NM_032223.1	SF3A1	0.42±0.04

Differential gene expressions between the KBD and normal samples were assessed using the selection criteria described in Materials and Methods. The public identification (ID) accession numbers refer to the numbers provided in the public databases RefSeq or GeneBank.

### qRT-PCR results of five selected genes

**Figure 2:**
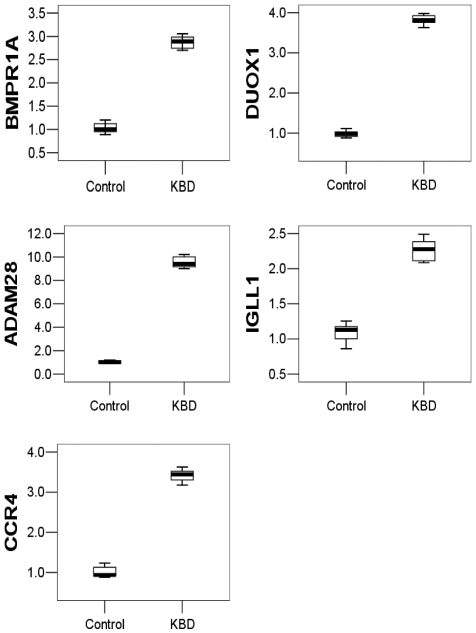
mRNA levels of the five selected genes (BMPR1A, DUOX1, ADAM28, IGLL1 and CCR4) in PBMCs of the controls and KBD patients. Steady-state mRNA levels were quantified by two-step SYBR Green real-time RT-PCR. The expression levels of the target genes in KBD PBMCs relative to those in control PBMCs were calculated using comparative Ct method. The lines inside the boxes denote the medians. The boxes mark the intervals between the 25th and 75th percentiles. The whiskers denote the intervals between the 10th and 90th percentiles.

Five of the differentially expressed genes identified in the microarray analysis were selected for qRT-PCR analysis using BPMC samples from additional 10 subjects to validate the oligonucleotide array data. We found that the mRNA expression levels of BMPR1A, DUOX1, ADAM28, IGLL1 and CCR4 genes in the KBD PBMCs and the normal control PBMCs were significantly different (Figure 2). More importantly, the changes were consistent with those revealed by the array analysis.

## Discussion

Focal chondronecrosis in the deep zones of articular cartilage and growth plate cartilage is a key feature of KBD. Over the past 50 years, low selenium and T-2 toxin contents in cereals from KBD areas in China have been investigated as the main risk factors for KBD, but little is known as to whether those environmental factors are linked to gene expressions and how they affect the pathogenesis of KBD. In this study, we presented evidence for differential gene expressions in the PBMCs of KBD patients. The obtained differentially expressed gene profile in the peripheral blood is expected to provide hints for the further study of pathogenesis of KBD and the establishment of early diagnostic criteria for KBD. Two main groups of up-regulated genes in KBD patients were identified: (1) Immunity-related genes, including IGLL1, IGHA1, and FREB. IGLL1 is critical for B-cell development. IGHA1 may serve to prevent access of foreign antigens to the general immunologic system in the human body. FREB mediates the antibody-induced destruction of IgG-coated antigens and of cells. The protein encoded by FREB is selectively expressed in B cells, and may be involved in their development. The protein may also be involved in the development of lymphomas. (2) Receptor-related genes, including CCR4 and BMPR1A. CCR4 is a receptor for chemokine and BMPR1A is a receptor for bone morphogenetic protein (BMP). CCR4 is expressed in human natural killer (NK) cells and involved in the regulation of the activation of chemokines such as macrophage-derived chemokine.

Five differentially expressed genes (BMPR1A, DUOX1, ADAM28, IGLL1 and CCR4) were selected for qRT-PCR assay because they might be related to the pathogenesis of KBD. CCR4, which belongs to the family of G-protein-coupled receptors, is a receptor for the CC chemokine. The ligands of CCR4 include CCL17, CCL22, MIP21A, RANTES, MCP-1 and TARC [12], [13]. Chemokines play fundamental roles in the development and function of the human immune system. IGLL1 is found on the surfaces of proB and preB cells, where it is involved in the transduction of signals for cellular proliferation and the differentiation at the proB cell to preB cell stage. Thus, IGLL1 is critical for B-cell development. The up-regulations of CCR4 and IGLL1 serve as indicators of immunity disorders in KBD patients. The KBD patients showed abnormal expressions of immunoglobulin-related genes in their PBMCs, indicating that immunological reactions may play an important role in the pathogenesis of KBD. It has been found that some compositions of KBD cartilage contain antigenic determinants [14], [15]. Moreover, chondrocytes also have the similar function as accessory cells in the immune process in that they provide tissue specific antigens, which may initiate or sustain autoimmune reactions [16]. The cellular immunity against the components of osteoarthritis (OA) cartilage has been reported in both OA animal models and OA patients [17]. Humoral immunity is also involved in the pathogenesis of OA [15]. Disorders in the humoral immunity and cellular immunity in the peripheral blood of KBD patients have been reported. Low levels of complement and degraded antibodies have been found in KBD children [18]. Hypofunction of the cellular immunity in KBD patients can lead to lower rosette forming and lymphocyte conversion ratios [19], and to skin erythema of shorter diameters [20]. All of these findings indicate that immunological reactions may play an important role in the pathogenesis of KBD. Furthermore, disorders in immunology may occur before radiological changes in KBD [21]. Therefore, the diagnostic criteria for KBD based on gene expression changes may help detect KBD in an earlier stage than the diagnostic criteria based on radiological changes.

Upregulation of DUOX1 in KBD patients indicates that reactive oxygen species (ROS) were increased in these patients since DUOX1 is an isoenzyme of nicotinamide adenine dinucleotide phosphate (NADPH)oxidases, one of its functions is to regulate the production of ROS. In addition, the increase in ROS may also be caused by the decreased selenium(Se) level, which has been found in the soil, wheat and corns from the KBD areas, as well as in the blood and hair of KBD children. Selenium as an essential trace element is involved in the functions of glutathione peroxidases (GPx) [22]. GPxs are antioxidant enzymes which protect membrane lipids and macromolecules against the oxidative damage induced by peroxides [23]. Most of the selenoproteins, including GPx isozymes, have ROS scavenging activities and the action of selenium has been known as an antioxidant system in cell survival^22^., Therefore, the production of ROS was increased in KBD patients most probably as a result of the up-regulation of DUOX1 and the down-regulation of antioxidant protection. ROS can mediate apoptosis by mitochondria dysfunction. Our recent study has shown that the mitochondrial function is altered in the articular chondrocytes of KBD patients [24]. Taken together, we can deduce that ROS may be involved in the pathogenesis of KBD.

BMPR1A belongs to the family of transmembrane serine/threonine kinases and its ligands are members of the transforming growth factor-beta (TGF beta) superfamily. TGF-betas and activins transduce their signals through the formation of a heteromeric complex of 2 different types of serine kinase receptors: TGF-beta type I receptor and TGF-beta type II receptor. BMPR1A is a main receptor for bone morphogenetic protein 2 (BMP-2), which plays an important role in the development of bone and cartilage. No previous study has shown the relationship between BMP-2 and KBD. However, our study shows that BMPR1A was up-regulated in KBD patients. BMPR1 and BMP-2 could be the next targets in KBD studies.

ADAM28 was found to be up-regulated in KBD patients. ADAM28 is a member of the ADAM (a disintegrin and metalloprotease domain) family. ADAM28 induced by all-trans retinoic acid can degrade both bovine and human proteoglycans in chondrocytes [25], which suggests that ADAM28 may be a new aggrecanase. Given that chondronecrosis in the deep zone of articular cartilage and growth plate cartilage is the key feature of KBD, ADAM28 may play a role in cartilage degradation of KBD patients.

A large number of genes have been reported to be involved in arthritis. Genes involved in the regulation of immune cell functions, receptor signaling as well as protein metabolism and degradation have been found to be up-regulated in juvenile arthritis [26]. Macrophage differentiation markers MNDA, MRP8 and MRP14, signaling molecules JAK3 and MAP kinase p38, receptors TNFR2/p75, CCR1 and CXCR4, integrin beta1, and the cytokines/chemokines interleukin (IL) 1beta and IL-8 have also been identified in patients with arthritis [27]. HSP90A1, IKBKAP, IL13RA1, CXCL14 and NFFAIP6 have been found to be significantly down-regulated in mild OA [28]. KBD is a specific type of osteoarthritis; however, there is relatively little overlap of the identified differential gene expressions between KBD and osteoarthritis. This difference may be caused by the incorrect expectation that the gene expression in arthritis will differ substantially from that in KBD, the different technical platforms used (Agilent GeneChip versus the Affymetrix GeneChip), the different array versions used, and the inherent differences among individuals.

PBMCs were used in this study because they are easy to collect, as compared with articular cartilage, which is usually isolated by biopsy or surgical operation. PBMCs are a good source for discovering biomarkers for KBD, especially in its early phase. In addition, blood is a highly dynamic environment, in which blood cells have a rapid natural turnover. Blood communicates with practically every tissue in the body, and is thus proposed as a ‘sentinel tissue’ that reflects disease progression in the body [29]. Thus, changes of gene expressions in PBMCs can help ease the preclinical and clinical drug development [30]. This strategy has also been successfully applied to the investigations of cancer biology [31], autoimmune disease [32], and cardiovascular disease [33]. This study showed that gene expression profiling in peripheral blood holds great promise for the development of clinical gene biomarkers specific to KBD [34].

In conclusion, KBD is associated with a distinct gene expression profile and a suppressed state of immunity in PBMC. The suppressed immunity may play an important role in the pathogenesis of KBD. It will be of great interest to extend these findings to address their applications to the identification of KBD patients and the prediction of the patients' responses to therapies at the earliest time-point possible.

## Supporting Information

Table S1 **List of selected up-regulated genes in KBD PBMC.** Differential gene expressions between the KBD and normal samples were assessed using the selection criteria described in Materials and Methods. The public identification (ID) accession numbers refer to the numbers provided in the public databases RefSeq or GeneBank.
(DOC)
