# Supplementary material for: Genome-Wide Gene Expression Analysis Suggests an Important Role of Suppressed Immunity in Pathogenesis of Kashin-Beck Disease
Source: PLoS One. 2012 Jan 3;7(1):e28439. doi: 10.1371/journal.pone.0028439 (PMC3250390; doi:10.1371/journal.pone.0028439)
Supplement: Table S1 — List of selected up-regulated genes in KBD PBMC. Differential gene expressions between the KBD and normal samples were assessed using the selection criteria described in Materials and Methods. The public identification (ID) accession numbers refer to the numbers provided in the public databases RefSeq or GeneBank. (DOC) [file pone.0028439.s001.doc]

**Table S1. List of selected up-regulated genes in KBD PBMC**

| Category | Gene name | Public ID | Gene symbol | Fold change Mean±SEM |
| --- | --- | --- | --- | --- |
| Immunity related | Immunoglobulin lambda-like polypeptide 1 | BC012293.1 | IGLL1 | 3.34±0.98 |
|  | Iimmunoglobulin heavy chain constant region alpha 1 | BC032249.1 | IGHA1 | 3.20±1.09 |
|  | Myomesin (M-protein) 2 | X69089.1 | MYOM2 | 3.15±1.07 |
|  | Fc receptor homolog expressed in B cells | AF531423.1 | FREB | 2.28±0.08 |
| Receptor | Bone morphogenetic protein receptor (type IA) | BC028383.1 | BMPR1A | 2.99±0.85 |
|  | Chemokine (C-C motif) receptor 4 | X85740.1 | CCR4 | 3.08±0.49 |
| Metabolism | Dual oxidase 1 | AF213465.1 | DUOX1 | 5.10±1.95 |
|  | Ectonucleoside triphosphate diphosphohydrolase 1 | NM_001776.2 | ENTPD1 | 2.66±0.37 |
|  | Stress 70 protein chaperone | BC036370.1 | STCH | 2.66±0.24 |
| Apoptosis | Baculoviral IAP repeat-containing 7 | BC014475.1 | BIRC7 | 2.39±0.18 |
|  | Tumor necrosis factor receptor superfamily member 6b | NM_032957.1 | TNFRSF6B | 2.24±0.06 |
| Cystoskeleton and cell movement | Homo sapiens chromosome 22 open reading frame 3 | AL050346.1 | C22orf3 | 2.61±0.27 |
|  | Triadin | NM_006073.1 | TRDN | 2.63±0.58 |
| DNA modification | Guanine deaminase | AB033084.1 | GDA | 7.71±3.21 |
|  | Homo sapiens polymerase (DNA directed) iota (POLI) | NM_007195.1 | POLI | 2.49±0.15 |
| Oncogene related | Sarcospan | AL136756.1 | SSPN | 2.59±0.30 |
|  | Cutaneous T-cell lymphoma (CTCL)-associated antigen 1 | AF177229.1 | CTAGE-1 | 2.56±0.42 |
| Protein synthesis and modification | Werner helicase interacting protein | AF218313.1 | WHIP | 4.19±1.94 |
|  | Protein containing six Kelch motifs and a BTB (BR-C, ttk and babor) or POZ (Pox virus and zinc finger) domain | NM_152467.1 | FLJ32662 | 2.71±0.69 |
| Development | Fragile X mental retardation autosomal homolog 1, | U25165.1 | FXR1 | 2.43±0.42 |
|  | Homo sapiens frizzled homolog 5 (Drosophila) (FZD5) | NM_003468.2 | FZD5 | 2.39±0.23 |
| Ion channel transport protein | Transmembrane gamma-carboxyglutamic acid protein 4 | BC010052.1 | TMG4 | 3.37±1.14 |
|  | Stomatin, human EPB72 | BC025760.1 | STOML3 | 2.41±0.22 |
|  | Homo sapiens potassium voltage-gated channel | NM_004732.2 | KCNAB3 | 2.37±0.07 |
|  | Homo sapiens potassium channel, subfamily K, member 3 (KCNK3) | NM_002246.1 | KCNK3 | 2.37±0.36 |
| Extracellular matrix related | Retired, was Disintegrin and metalloproteinase domain 28 | NM_021777.1 | ADAM28 | 3.37±0.43 |
|  | Hair keratin 6 (type II) | X99142.1 | KRTHB6 | 2.39±0.33 |
|  | Homo sapiens keratin associated protein 9-9 (KRTAP9-9) | NM_030975.1 | KRTAP9-9 | 2.31±0.05 |
| Cytokine factor | Protein with high similarity to elongation of very long chain fatty acids-like 3 | BC034344.1 | ELOVL3 | 2.27±0.12 |
|  | Signal sequence receptor gamma | BC017203.1 | SSR3 | 2.26±0.18 |
| Cell cycle | Cyclin B1, regulatory subunit of the CCNB1 | BC006510.1 | CCNB1 | 2.94±0.91 |
|  | Rad50-interacting protein 1 | NM_021930.2 | FLJ11785 | 2.89±0.68 |
| Zinc finger protein | Zinc finger protein 25 | NM_016220.1 | ZFD25 | 2.82±0.77 |
|  | Zinc finger protein 195 | NM_007152.1 | ZNF195 | 2.72±0.36 |
| Signal transduction | Protein containing three WD domains (WD-40 repeats) | NM_018051.2 | FLJ10300 | 2.63±0.42 |
|  | Cerebral cavernous malformations 1 | NM_004912.1 | CCM1 | 2.47±0.23 |
| Transcription related | Protein with high similarity to zinc finger protein 234 | NM_024733.1 | FLJ14345 | 11.38±6.21 |
|  | Homo sapiens chromosome 21 open reading frame 81 (C21orf81) | NM_153750.1 | C21orf81 | 2.80±0.62 |
|  | Suppressor of var1 S | BC036112.1 | SUPV3L1 | 2.78±0.54 |
|  | POU domain class 4 transcription factor 1 | L20433.1 | POU4F1 | 2.72±0.63 |
| Miscellaneous | Protein of unknown function, has strong similarity to uncharacterized mouse AF064782 | NM_032042.2 | DKFZP  564D172 | 3.12±1.02 |
|  | Homo sapiens hypothetical protein MGC26568 | NM_152402.1 | TRAM1L1 | 3.12±1.03 |
|  | Protein of unknown function | NM_032811.1 | TBRG1 | 2.72±0.62 |
|  | Protein of unknown function | NM_018616.1 | PRO2037 | 2.58±0.37 |
|  | Member of the uncharacterized RIO1-ZK632.3-MJ0444 family | AK002021.1 | FLJ11159 | 2.49±0.28 |

Differential gene expressions between the KBD and normal samples were assessed using the selection criteria described in Materials and Methods. The public identification (ID) accession numbers refer to the numbers provided in the public databases RefSeq or GeneBank.
